# Exploitation of Herpesvirus Immune Evasion Strategies to Modify the Immunogenicity of Human Mesenchymal Stem Cell Transplants

**DOI:** 10.1371/journal.pone.0014493

**Published:** 2011-01-06

**Authors:** Anabel S. de la Garza-Rodea, Marieke C. Verweij, Hester Boersma, Ietje van der Velde-van Dijke, Antoine A. F. de Vries, Rob C. Hoeben, Dirk W. van Bekkum, Emmanuel J. H. J. Wiertz, Shoshan Knaän-Shanzer

**Affiliations:** 1 Department of Molecular Cell Biology, Leiden University Medical Center, Leiden, The Netherlands; 2 Department of Medical Microbiology, Leiden University Medical Center, Leiden, The Netherlands; 3 Department of Medical Microbiology, University Medical Center Utrecht, Utrecht, The Netherlands; New York University, United States of America

## Abstract

**Background:**

Mesenchymal stem cells (MSCs) are multipotent cells residing in the connective tissue of many organs and holding great potential for tissue repair. In culture, human MSCs (hMSCs) are capable of extensive proliferation without showing chromosomal aberrations. Large numbers of hMSCs can thus be acquired from small samples of easily obtainable tissues like fat and bone marrow. MSCs can contribute to regeneration indirectly by secretion of cytokines or directly by differentiation into specialized cell types. The latter mechanism requires their long-term acceptance by the recipient. Although MSCs do not elicit immune responses *in vitro*, animal studies have revealed that allogeneic and xenogeneic MSCs are rejected.

**Methodology/Principal Findings:**

We aim to overcome MSC immune rejection through permanent down-regulation of major histocompatibility complex (MHC) class I proteins on the surface of these MHC class II-negative cells through the use of viral immune evasion proteins. Transduction of hMSCs with a retroviral vector encoding the human cytomegalovirus US11 protein resulted in strong inhibition of MHC class I surface expression. When transplanted into immunocompetent mice, persistence of the *US11*-expressing and HLA-ABC-negative hMSCs at levels resembling those found in immunodeficient (i.e., NOD/SCID) mice could be attained provided that recipients' natural killer (NK) cells were depleted prior to cell transplantation.

**Conclusions/Significance:**

Our findings demonstrate the potential utility of herpesviral immunoevasins to prevent rejection of xenogeneic MSCs. The observation that down-regulation of MHC class I surface expression renders hMSCs vulnerable to NK cell recognition and cytolysis implies that multiple viral immune evasion proteins are likely required to make hMSCs non-immunogenic and thereby universally transplantable.

## Introduction

Mesenchymal stem cells (MSCs) are multipotent cells present in the stroma of most organs. Their isolation relies on adherence to cell culture plastics. In culture, human MSCs (hMSCs) are capable of extensive proliferation without giving rise to chromosomal aberrations [1]. Large numbers of hMSCs can thus be obtained from small samples of easily accessible tissues like fat and bone marrow (BM).

MSCs are identified primarily on the basis of their ability to differentiate under specific culture conditions into various mesodermal cell types including osteoblasts, chondrocytes and adipocytes. Since markers exclusively displayed on the surface of MSCs have not been identified yet, these cells are phenotypically characterized by a combination of several non-hematopoietic cell surface markers [2].

Typically, cultured hMSCs express on their surface the major histocompatibility complex (MHC) class I human leukocyte antigens A, B, C (HLA-ABC) and HLA-G, but not HLA-E 3, 4 and our unpublished observations. Furthermore, hMSCs do not display MHC class II (HLA-DR) proteins on their plasma membrane. MHC class II molecules are present, however, in intracellular reservoirs and can be recruited to the cell surface through exposure to interferon gamma (IFN-γ) [5], [6].

Recently, cultured MSCs were shown to have pleiotropic immunomodulatory properties *in vitro* including suppression of T cell proliferation in response to alloantigens or mitogens [7], [8], inhibition of B cell proliferation and antibody production [9], [10] and inhibition of dendritic cell maturation [11], [12]. Also, cultured hMSCs are not lysed by freshly isolated allogeneic natural killer (NK) cells but are susceptible to the lytic activity of activated NK cells [13], [14]. *In vivo*, allogeneic MSCs were shown to prolong the survival of skin allografts in baboons [7] and hMSCs ameliorated experimental autoimmune encephalitis in mice [15]. In patients, infusion of allogeneic hMSCs has been shown to mitigate acute graft-versus-host disease to various degrees [16]–[18]. The reputation “non-immunogenic” that has been bestowed upon MSCs on the basis of these findings, has been challenged by studies with laboratory animals showing rejection of MSCs in allogeneic transplantation settings. The therapeutic benefits that have been observed after *in vivo* administration of MSCs are thus commonly believed to result mainly or exclusively from paracrine effects [reviewed in 19].

Repair of tissue damage that requires *in situ* differentiation of MSCs into specialized cell types or their fusion with resident cells has been attained only with autologous/syngeneic MSCs or in immunocompromised recipients [20]–[25]. Similarly, successful use of MSCs as vehicles for the delivery of therapeutics depends on immunocompatible donor-recipient combinations [26], [27].

The involvement of surface-displayed MHC class I molecules in graft rejection and the mitigation of transplant immunogenicity through interference with MHC class I protein recognition have been well documented. Masking of MHC class I molecules by specific antibodies enabled transplantation of human pancreatic islands and liver cells in mice and of porcine neurons in rats [28], [29]. Moreover, neurons of MHC class I^−^ transgenic mice were not rejected in rats [30]. Along the same line, adipose tissue-derived hMSCs that had lost MHC class I surface expression during long-term culture, effectively contributed to skeletal muscle repair in immunocompetent dystrophic mice [31]. Recently, Zdoroveac and co-workers [32] demonstrated reduced immune responses to carotid allografts genetically modified to decrease surface levels of MHC class I antigens through an endoplasmic reticulum-targeted MHC class I-specific intrabody.

Inhibition of MHC class I surface expression is a mechanism evolved by viruses to prevent killing of their targets cells by the hosts' immune system [33], [34]. Examples are herpesviruses that encode so-called immune evasion proteins (also known as immunoevasins), which specifically target different steps of the MHC class I-mediated peptide presentation pathway to elude the activity of CD8^+^ T cells. Some of these proteins, like the bovine herpesvirus type 1 (BHV-1) UL49.5 protein and the Epstein-Barr virus (EBV) BNLF2a protein, are inhibitors of the transporter associated with antigen processing (TAP), an essential component of the MHC class I antigen presentation pathway [35]–[37]. Other herpesviral proteins like the human cytomegalovirus (HCMV) *US2* and *US11* gene products, target MHC class I molecules for destruction through dislocation of newly synthesized proteins into to the cytosol where they are degraded by proteasomes [38], [39]. Herpesviruses also evolved strategies to interfere with the presentation of viral antigens to MHC class II-restricted CD4^+^ T cells and to escape NK cell responses reviewed in 40, 41.

In this study, we investigated whether immune rejection of foreign cells could be prevented by controlled permanent down-regulation of MHC class I surface expression. Using retroviral vectors (RVs) encoding four different herpesviral immunoevasins, we identified the US11 protein as a very effective inhibitor of MHC class I surface display in hMSCs. The immunogenicity of MHC class I^−^ hMSCs should ideally have been tested in an allogeneic recipient. This not being feasible, we resorted to the use of mouse models to study the *in vivo* persistence of hMSCs displaying normal or greatly reduced numbers of MHC class I molecules at their plasma membrane. In this xenotransplantation setting, we found *US11*-transduced hMSCs to be protected from rejection in immunocompetent recipients, albeit only after depletion of NK cells. This is, to our knowledge, the first *in vivo* study demonstrating the utility of herpesviral immunoevasins to modulate the immunogenicity of transplanted culture-expanded primary human cells.

## Results

### Herpesviral immune evasion proteins greatly differ in their ability to inhibit HLA-ABC expression on the surface of hMSCs

**Figure 1:**
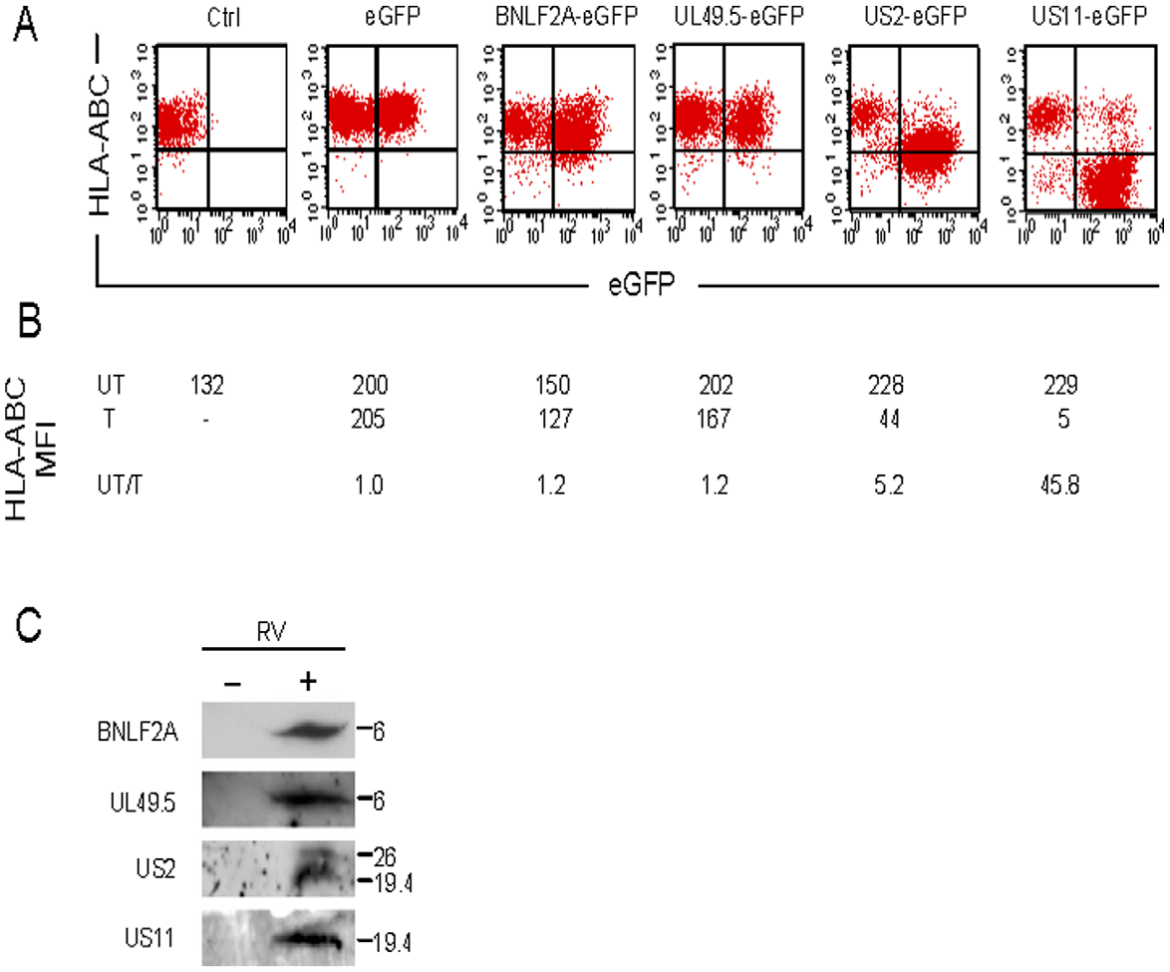
Different herpesviral immunoevasins inhibit MHC class I expression on hMSCs to a different extent. (A) Flow cytometric analysis of culture-expanded (Ctrl) hMSCs and of hMSCs transduced with RVs coding for eGFP alone or for eGFP and either the BHV-1 UL49.5, EBV BNLF2A, HCMV US2 or HCMV US11 protein. The data are from cells analyzed 30 days after transduction. Analysis of the hMSCs at 5 and 90 days post-transduction yielded very similar results (data not shown). MFIs of the HLA-ABC signal of eGFP^-^ (untransduced [UT]) and eGFP^+^ (transduced [T]) cell populations in each sample and their ratio are given below the dot plots (B). (C) Western blot analysis of lysates from untransduced hMSCs (−) and of hMSCs transduced with RV-BLF2A-eGFP, RV-UL49.5-eGFP, RV-US2-eGFP or RV-US11-eGFP (+). The specificity of the antibodies used is indicated at the left. Numbers at the right represent molecular weights in kilodalton (kDa). The 26-kDa and 19.4-kDa protein species recognized by the US2-specific antibodies correspond to N-glycosylated and non-glycosylated forms of the US2 protein, respectively.

Four different herpesviral immunoevasins were tested for their ability to alter the expression of HLA-ABC on the surface of hMSCs. To this end, hMSCs from a single donor (i.e. donor 1) were stably transduced with bicistronic RVs coding for the enhanced green fluorescent protein (eGFP) and for the product of the BHV-1 *UL49.5* gene (RV-UL49.5-eGFP), the EBV *BNLF2a* gene (RV-BNLF2a-eGFP), the HCMV *US2* gene (RV-US2-eGFP) or the HCMV *US11* gene (RV-US11-eGFP). Untransduced hMSCs and cells transduced with an RV coding for eGFP only (RV-eGFP) served as negative controls. Flow cytometric analysis performed 5, 30 and 90 days after transduction revealed that a large proportion of the cells in each of the transduced samples expressed *eGFP* (Fig. 1A). The HLA-ABC levels on the plasma membrane of the eGFP^+^ cells, as determined by staining with HLA-ABC-specific antibodies differed, however, depending on the herpesviral gene present (Fig. 1A). Whereas untransduced and RV-eGFP-transduced hMSCs expressed high levels of plasma membrane-bound MHC class I molecules, HLA-ABC molecules were barely detectable on the surface of cells transduced with RV-US11-eGFP. A comparison of the HLA-ABC signal intensities between the GFP^+^ and GFP^−^ (i.e. untransduced) cells in the RV-US11-eGFP treated sample (Fig. 1B) showed that forced *US11* expression causes a 46-fold reduction in HLA-ABC surface levels (mean fluorescent activity [MFI] of 5 and 229, respectively). Down-regulation of plasma membrane-bound HLA-ABC molecules was also observed in hMSCs exposed to RV-US2-eGFP albeit to a lesser extent (with a MFI ratio of untransduced versus transduced cells of 5.2). In hMSCs transduced with the RVs encoding the BNLF2a or UL49.5 protein, HLA-ABC surface expression was reduced only marginally (for both vectors the MFI ratios of untransduced versus transduced cells was 1.2).

The similar frequency of eGFP^+^ cells and the similar average intensity of the eGFP signal in the eGFP^+^ cells in all RV-treated samples suggest that hMSCs had been transduced with approximately equal efficiency by the different RVs. Western blot analysis of proteins extracted from hMSCs at 21 days after transduction confirmed the presence of the UL49.5, BNLF2a, US2 and US11 proteins (Fig. 1C).

**Figure 2:**
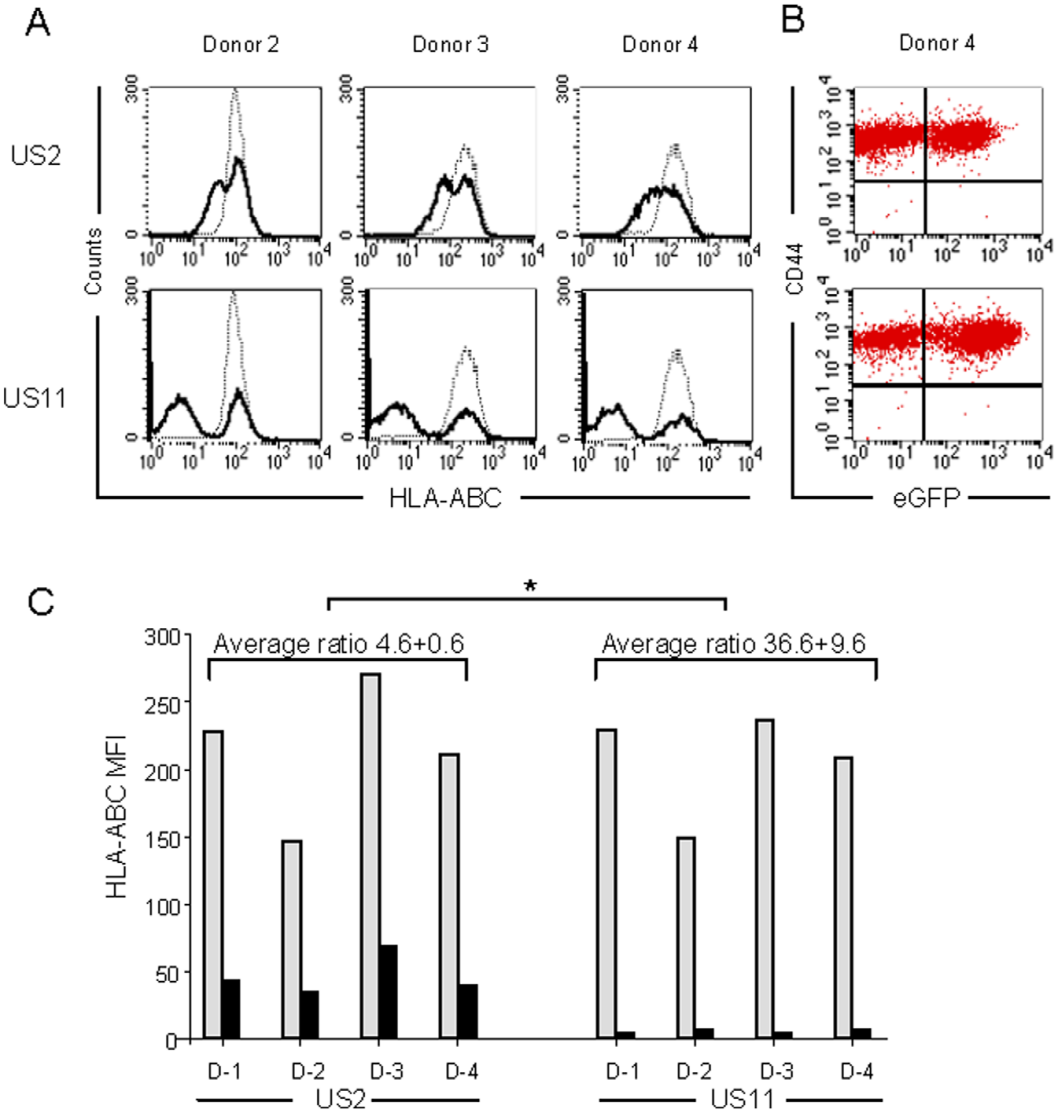
US2 and US11 inhibit MHC class I surface expression in hMSCs of different donors. (A) Flow cytometric analysis of HLA-ABC surface expression in hMSCs derived from the BM of three different donors. Gray lines represent untransduced hMSCs. Black lines correspond to hMSCs transduced with RV-US2-eGFP (upper panels) or with RV-US11-eGFP (lower panels). The presented data are from cells analyzed 30 days after transduction. (B) Flow cytometric analysis of CD44 surface expression in hMSCs of donor 4 at 30 days after transduction with RV-US2-eGFP (upper panel) or RV-US11-eGFP (lower panel). Similar results were obtained with cells of all other donors (data not shown). (C) Comparison of down-regulation of HLA-ABC surface expression by RV-US2-eGFP and RV-US11-eGFP. Shown are HLA-ABC MFI values of untransduced (gray bars) and transduced (black bars) cells of single samples of culture-expanded hMSCs from 4 donors (marked D1 to D4; values derived from data presented in Fig. 1 and 2). The differences in the average MFI ratio (calculated as in Fig. 1) between the hMSCs exposed to RV-US2-eGFP and those transduced with RV-US11-eGFP is highly significant (*p = 0.0005).

Transduction of hMSCs isolated from the BM of three additional donors (i.e. donors 2, 3 and 4) with RV-US2-eGFP or RV-US11-eGFP yielded very similar results as for donor 1 (data presented in Fig. 2A and 2C). All cells with down-modulated HLA-ABC surface expression were also eGFP^+^. Furthermore, transduction with the RVs did not affect plasma membrane levels of the CD44 protein, a marker of hMSCs (Fig. 2B).

### Persistence of herpesviral immune evasion proteins in hMSCs does not compromise their replication capacity

**Figure 3:**
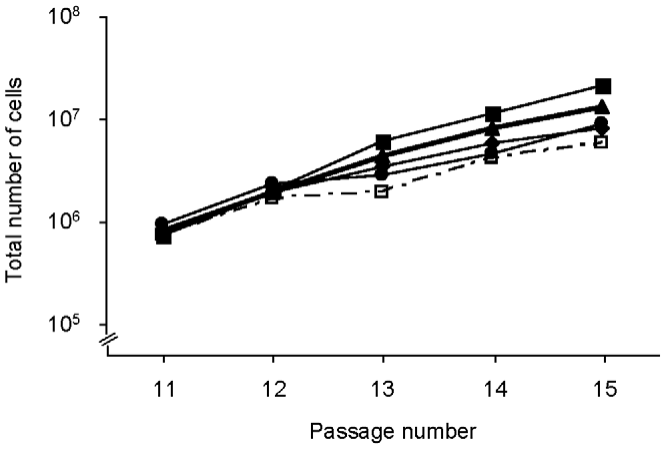
Herpesviral immunoevasins do not compromise the replication rate of hMSCs in culture. hMSCs were transduced at passage number 8 with bicistronic RVs encoding eGFP and a herpesviral immunoevasin and kept in culture for 7 additional passages. Lines represent untransduced hMSCs (□) and cells transduced with RV-UL49.5-eGFP (♦), RV-BNLF2A-eGFP (▴), RV-US2-eGFP (▪) or RV-US11-eGFP (•) during 5 passages.

hMSCs transduced to express either of the four herpesviral *immunoevasin* genes were kept in culture for five additional passages. At each passage, the number of cells was determined and cell aliquots were analyzed for the presence of eGFP and surface HLA-ABC. Overall growth characteristics were not affected by the expression of the herpesviral *immunoevasin* genes (Fig. 3).

During the entire period of analysis, the frequency of eGFP^+^ cells, the average eGFP signal per eGFP^+^ cell, as well as the cell surface expression level of HLA-ABC remained constant (data not shown).

### IFN-γ partially overrules US2- and US11-mediated down-regulation of MHC class I surface expression and elevates surface levels of MHC class II molecules

**Figure 4:**
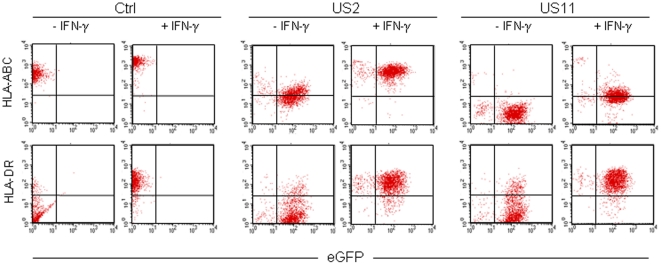
IFN-γ modulates expression of MHC class I and class II molecules on US2- and US11-transduced hMSCs. Flow cytometric analysis of the expression of MHC class I (HLA-ABC; upper panels) or MHC class II (HLA-DR; lower panels) proteins on the surface of untransduced hMSCs (Ctrl) and of cells transduced with RV-US2-eGFP (US2) or with RV-US11-eGFP (US11). The cells were (+ IFN-γ) or were not (- IFN-γ) incubated for 48 hours with 100 ng/ml IFN-γ prior to flow cytometry. The *US11*- and *US2*-transduced hMSCs used for this experiment, were sorted on the basis of *eGFP* expression.

Previous studies have demonstrated that treatment of hMSCs with IFN-γ induces cell surface expression of MHC class II molecules [5], [6]. We confirmed this finding (Fig. 4) with untransduced hMSCs and showed that the same applies to hMSCs transduced with RV-US2-eGFP or RV-US11-eGFP. In all three cell populations surface MHC class II levels were equally high after incubation for 48 hours with 100 ng/ml of IFN-γ. Surface MHC class I expression after IFN-γ treatment depended on the levels of plasma membrane-bound MHC class I molecules prior to cytokine stimulation, i.e. HLA-ABC surface expression in RV-US2-eGFP-transduced hMSCs was similar to that in untransduced cells not treated with IFN-γ but it was only partially restored in cells transduced with RV-US11-eGFP.

### Persistence of HLA-ABC-negative hMSCs in immunocompetent mice requires depletion of NK cells

The effect of MHC class I surface expression on the engraftment of hMSCs in mice was addressed by comparing the persistence of RV-US11-eGFP-transduced hMSCs (US11-hMSCs) with that of unmodified cells after intrapinnal implantation into immunodeficient or immunocompetent mice.

**Figure 5:**
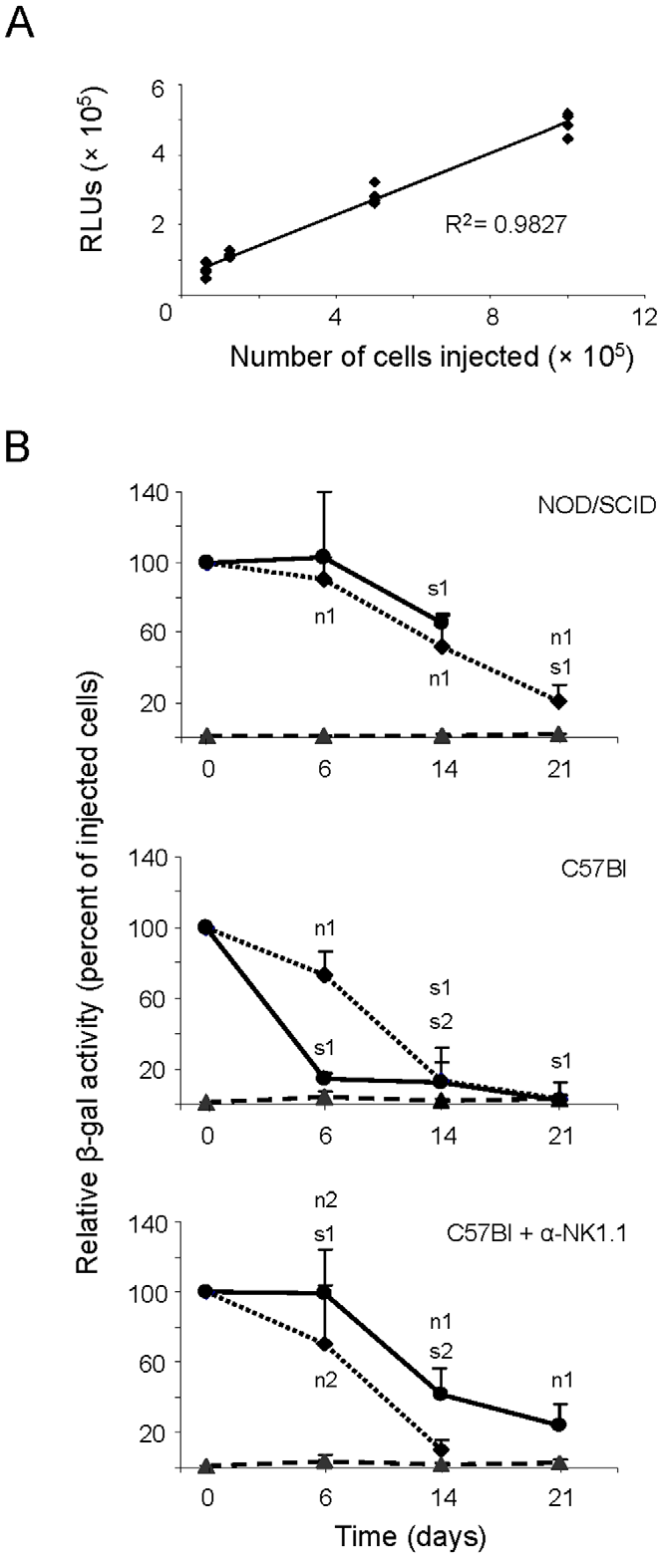
Engraftment of hMSCs with modulated surface MHC class I expression in immunodeficient and immunocompetent mice. (A) Correlation between donor cell number and β-gal activity (expressed in RLUs) in the ears of mice following intrapinnal injection of *LacZ*-expressing hMSCs. The ears of the treated mice (four mice for each cell dose) were excised 5 minutes after cell injection and processed as described in the Materials and Methods section. Each dot represents a single ear. (B) Engraftment of hMSCs in immunodeficient mice (upper graph), immunocompetent mice (middle graph) and immunocompetent mice depleted of NK cells (lower graph). Indicated on the vertical axis is the average β-gal activity in whole ears of mice sacrificed at the specified time points after the injection of cell or PBS as compared to that of mice killed 5 minutes after the injection of cells from the same batch (time 0; 100%). The graphs show time-related changes in relative β-gal activity for sorted US11-hMSCs (solid line and •) and for untreated hMSCs (dotted line and ♦) in NOD/SCID mice, C57Bl mice and NK cell-depleted C57Bl mice. Background β-gal activity values were derived from mice whose pinnae were injected with vehicle only (broken line and ▴). Individual data points represent the average (± SD) relative β-gal activity of 3 to 14 animals and have been derived from 2 to 4 independent experiments. Statistical analysis was performed at each time point and through all three graphs for pairs of data points derived from all mice in a particular experimental group. Significant difference (p<0.002) between data points were marked s1 and s2 while non-significant differences (p>0.5) between data pairs are indicated by n1 and n2.

To allow quantification of the surviving donor cells, we used in this study US11-hMSCs and control hMSCs that were endowed with a recombinant *LacZ* gene by transduction with the self-inactivating lentiviral vector LV.C-EF1a.cyt-bGal. The β-galactosidase (β-gal) activity in treated ears was determined with the Beta-Glo assay system. Validation of this assay system revealed a linear correlation between β-gal activity (expressed in relative light units [RLUs]) and the number of donor cells injected (Fig. 5A).

In the immunodeficient NOD/SCID mice, the survival of control (i.e. HLA-ABC^+^) hMSCs and US11-hMSCs was similar during the first 14 days after implantation with a reduction in β-gal activity to 52±19 and 65±4% of day 0 values, respectively (Fig. 5B, top panel).

In the immunocompetent C57Bl mice, the decrease in the number of unmodified (i.e. HLA-ABC^+^) hMSCs was more rapid, i.e. on average only about 10% of the input cells remained at two weeks after transplantation (Fig. 5B, middle panel). This difference in the rate of reduction in β-gal activity between the two mouse strains is considered to reflect immune rejection of the hMSCs. Interestingly, following implantation in immunocompetent recipients the US11-hMSCs disappeared faster than the unmodified hMSCs (Fig. 5B, middle panel).

**Figure 6:**
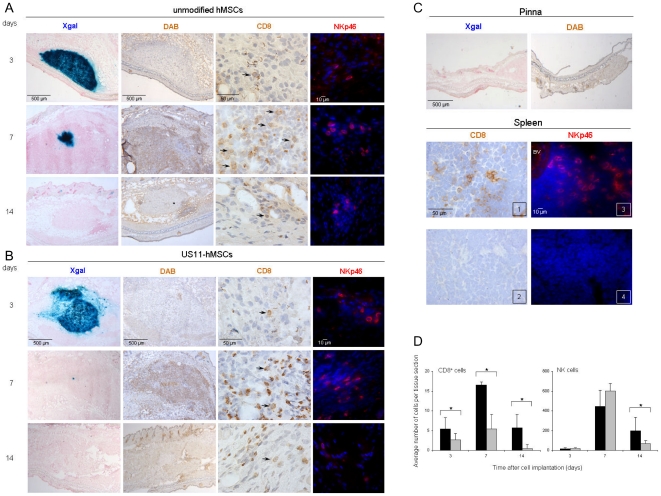
Infiltration of recipient's immune cells in the vicinity of the hMSC-implants. Immunohistological and histochemical analysis of frozen tissue section. (A and B) Pinnea excised 3, 7 and 14 days after implantation of *LacZ*-transduced hMSCs (A) or *LacZ*-transduced US11-hMSCs (B). Implanted hMSCs are visualized in the pinnea by X-gal (blue) staining. DAB staining was used to identify granulocytes (endogenous peroxidase) and CD8^+^ cells. The granulocytes are recognized by the intracellular localization of the brown precipitate while the CD8^+^ cells (arrows) are predominantly showing staining of the plasma membrane. The anti-NKp46 antibody was used to identify NK cells (red, cell membrane staining) and nuclei were stained with Hoechst 33342 (blue). (C) Control tissues (pinna and spleen). (D) CD8^+^ and NK cell counts in pinnea transplanted with untreated hMSCs (black bars) or US11-hMSCs (gray bars). The data represent averages ± SD of CD8^+^ and NKp46^+^ cells present in 9 different tissue sections (3 pinnea per experimental group and 3 sections with a mutual distance of 32 µm per pinna). *p<0.05.

Immunohistological analysis of pinnea at 3, 7 and 14 days after implantation of unmodified hMSCs or US11-hMSCs revealed little cell infiltration at day 3 but massive infiltration of granulocytes and NK cells at day 7, regardless of the MHC class I surface levels of the implanted cells (Fig. 6). At day 14 after transplantation, only few granulocytes and NK cells were detected in the pinnea (Fig. 6A, 6B and 6D). The number of infiltrating CD8^+^ cells was, at all time points significantly higher in pinnea implanted with unmodified hMSCs than in those injected with US11-hMSCs (Fig. 6D).

**Figure 7:**
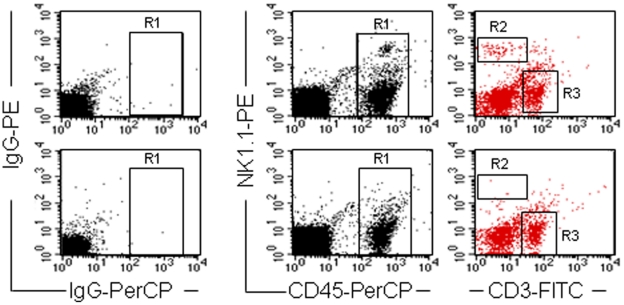
NK cell levels in C57Bl mice treated with NK1.1-specific MAbs. Flow cytometric analysis of PBMCs of a representative mouse stained with antibodies directed to murine CD45, CD3 and NK1.1. The upper panels show data of peripheral blood taken before treatment with NK1.1-specific MAbs. The lower panels are derived from a peripheral blood sample taken 7 days after intraperitoneal injection of 100 µg anti-NK1.1 antibodies. Cells stained with isotype-matched control antibodies are depicted in the left column. In the right column only CD45^+^ cells that were gated in R1 (middle column) have been analyzed for surface expression of NK1.1 (gate R2) and CD3 (gate R3). All peripheral blood samples of mice treated with NK1.1-specific MAbs showed similar low levels of NK1.1^+^ CD3^−^ cells.

To visualize NK cells, consecutive tissue sections were stained with either anti-NKp46, anti-Ly49G2 or anti-NK1.1 antibodies. Although NK.1.1^+^CD3^−^ and NK1.1^+^CD3^+^ cells were identified by flow cytometry among peripheral blood mononuclear cells (PBMCs) of C57Bl mice (Fig. 7), we could not detect NK1.1^+^ cells in sections of pinnea or control tissues (spleen and lymph nodes, data not shown). Accordingly, in Fig. 6 sections stained with the NKp46-specific antibody are presented. Similar frequencies and distributions of positive cells were found in the sections stained with the anti-Ly49G2 antibody (no statistical significant difference, data not shown). Ly49G2 is present on the surface of both NK and NK T cells, in contrast to the NKp46 protein, whose surface expression is essentially restricted to NK cells. Our finding may thus indicate the absence of substantial numbers of NK T cells in the vicinity of the grafted cells.

The knowledge that lack of classical MHC class I surface molecules can render cells susceptible to NK cell-mediated lysis [42], and the evident infiltration of NK cells into the implantation site, led to the proposition that NK cells are involvement in the early rejection of US11-hMSCs in C57Bl mice.

Indeed, C57Bl recipient mice depleted of NK cells through repeatedly administration of NK1.1-specific antibodies (Fig. 7) maintained US11-hMSCs implants at levels similar to those in NOD/SCID mice, which naturally display low NK cell activity (Fig. 5B, lower panel). NK cell depletion did not significantly affect the rate of decline of unmodified hMSCs in C57Bl mice. The difference in the rejection kinetics of US11-hMSCs between C57Bl mice with and without NK cells signifies that US11-hMSCs can trigger NK cell activation *in vivo* and serve as targets for their cytolytic activity.

## Discussion

Modulation of immunogenicity using viral immune evasion strategies has become a field of active research over the past decade. *In vitro* studies conducted primarily with establish cell lines revealed efficient inhibition of MHC class I/II surface expression after transduction with viral vectors encoding EBV immunoevasins. We show here that of four different herpesviral immunoevasins previously reported to interfere with the MHC class I antigen-presenting pathway, only the HCMV US11 protein strongly downregulates MHC class I expression on the surface of culture-expanded primary hMSCs. The HCMV US2 protein, which like the US11 protein, dislocates class I heavy chain molecules into the cytosol for subsequent degradation by proteasomes [39], was less effective in the hMSCs. Using adenoviral vectors, Rehm et al. [43] found that in primary human dendritic cells surface MHC class I expression was also suppressed much more efficiently by the US11 protein as compared to the US2 protein while in the human astrocytoma cell line U373 MG both immunoevasins were highly effective. Comparison of the amounts of the US11 and US2 proteins in the two cell types made the authors conclude that the differential effects of these two immunoevasins on the dendritic cells are not caused by the differences in their steady-state levels. Rather, studies with U373 MG cells expressing different murine class I heavy chain proteins [44] and J26 cells (i.e. human *B2M*-transduced murine fibroblasts) expressing distinct human HLA-ABC allomorphs [45], [46] revealed that US2- and US11-mediated down-regulation of MHC class I surface expression in these cell types is haplotype-specific. Notably, in our study, hMSCs derived from BM of four donors showed a very similar US2- and US11-dependent decrease in MHC class I surface expression.

The observed differences in the extent to which US2 and US11 inhibit expression of HLA-ABC on the surface of hMSCs may relate to the fact that these immunoevasins depend on distinct cellular proteins for their activity, which may be differently regulated in hMSCs. For example, US11-mediated degradation of MHC class I molecules requires Derlin-1 while the activity of US2 is Derlin-1-independent [47], [48]. Also, ubiquitination of MHC class I heavy chains is essential for their dislocation into the cytosol by US2 but not by US11 [49].

Whatever the underlying cause, the observed difference between distinct herpesviral immune evasion proteins in their capacity to suppress MHC class I expression on the surface of hMSCs argues for the screening of different immunoevasins for each specific target cell type, donor and application.

The utility of a herpesviral immunoevasin to inhibit hMSC rejection has been demonstrated here in a xenotransplantation model. Our results show that permanent interference with surface expression of MHC class I molecules with the aid of the US11 protein facilitates maintenance of the grafted cells in NK cell-depleted immunocompetent mice at levels similar to those obtained in immunodeficient recipients. The rapid decrease of the β-gal activity in the ears of immunocompetent mice following intrapinnal transplantation of *LacZ*-transduced US11-hMSCs, and its prevention by NK cell depletion points to NK cell-mediated rejection of the MHC class I^−^ donor cells in this animal model.

The kinetics of donor cell infiltration into the implanted pinnea, as observed in this study (Fig. 6), differs from the acute (within 24 hours) infiltration of innate immune cells into solid organ implants or into sites of cutaneous inflammation [50], [51]. We propose that this delay (few cells at day 3 post implantation and high numbers at day 7) is caused by the implanted hMSCs and in particular their ability to modulate the cytokine milieu near the site of cell injection. Implantation of hMSCs into ischemic kidneys resulted, after 24 hours, in an increase in anti-inflammatory cytokine (i.e IL-10) gene expression and in a decrease in mRNA levels for the pro-inflammatory cytokines IFN-γ, TNF-α and IL-1β [52]. Interestingly, in MSC-treated infarcted rat hearts pro/anti-inflammatory cytokine gene expression ratios increased between 24 hours and 2 weeks after MSC implantation [53]. This phenomenon may well explain the delayed leukocyte infiltration following hMSC implantation in our experiments.

The elimination of MHC class I^−^ hMSCs by NK cells would be consistent with the “missing self” hypothesis [54] and the reported prominent role of NK cells in xenograft rejection [55]. Our findings, however, do not concur with other studies reporting engraftment of MHC class I^−^ cells in xenogeneic or allogeneic recipients without active suppression of NK cell activity [28]–[32], [56]. In view of the different strategies used in these studies (i.e. antibody-mediated masking, *B2M* gene knockout, long-term culture, proteins encoded by the adenoviral early region 3) it is tempting to speculate that the observed differences result from qualitative and quantitative differences in residual HLA antigen expression on the surface of the donor cells. Also the donor cell type and the specific NK cell repertoire of the recipients may have affected the outcome of the experiments.

It is worth noting that hMSCs do not persist at constant high levels in immunodeficient mice. A time-related decline in the number of implanted hMSCs, as observed in this study, has also been documented in other xenograft models that enabled quantification of the implanted cells [57]–[59].

This disappearance of xenografted cells in the absence of adaptive immune responses is poorly understood. Involvement of host innate immune cells has been proposed but, so far, not proven conclusively [58], [60]. Alternatively, the observed decline may reflect an intrinsic property of implanted “free cells” that have been deposited in an unfavorable microenvironment. This perception is supported by results of Eliopoulos and colleagues, showing a limited persistence of murine MSCs following subcutaneous implantation into syngeneic recipients. Long-term survival of the implanted cells was significantly improved by embedding the MSCs into a matrix prior to implantation, emphasizing that the subcutis represents a suboptimal milieu for MSC transplantation [27], [61]. The observation that US11-hMSC numbers diminish in NK cell-depleted C57Bl mice with similar kinetics as in NOD/SCID mice suggests that *in vivo* these MHC-class I^−^ cells do not activate any other immune effector cells than the NK cells. This is especially interesting in view of our finding that like in *US11*-transduced tumor cell lines [62], *in vitro* exposure of US11-hMSCs to IFN-γ alleviates the effect of the immunoevasin and upregulates cell surface MHC class II protein expression. Apparently, such upregulation does not occur to any significant extent *in vivo*. Rather, a strong inhibition of the expression of genes encoding pro-inflammatory cytokines including IFN-γ was recorded in injured kidney tissue of rats following administration of MSCs [52]. If the interpretation of our results is correct, endowing hMSCs with the US11 protein and an immunoevasin that efficiently blocks NK cell-mediated cytolysis would result in completely non-immunogenic hMSCs for universal application.

Protection against the adverse effects of NK cells could be provided by viruses as well. Human and murine cytomegalovirus, for example, encode MHC class I-like molecules that can serve as decoys for NK cells [63], [64]. Other strategies to inhibit NK cell-mediated donor cell destruction including interference with their activation receptors and modulation of certain cytokine-dependent signaling pathways have also been described reviewed in 65.

In conclusion, we have demonstrated the potency of an HCMV immunoevasin to completely downregulate MHC class I expression on the surface of culture-expanded primary hMSCs. Following subcutaneous implantation into immunocompetent mice, the MHC class I^-^ hMSCs underwent fast rejection that was attributed to NK cell activity. However, in immunocompetent mice depleted of NK cells, US11-hMSCs gradually disappeared at a rate similar to that in immunodeficient hosts. Admittedly it is by no means certain that the xenograft model used in this study is fully predictable for the human situation, but our results show at least the expediency of viral immunoevasins in hMSCs and provide a rationale for transferring additional immunoevasin genes to generate universal donor cells.

## Materials and Methods

### Isolation and culture of BM-derived hMSCs

hMSCs were obtained from BM samples of four donors (2 males and 2 females, age range 38 to 72 years) collected during orthopedic surgery with a written informed consent and according to the guidelines of the Leiden University Medical Center (LUMC, Leiden, the Netherlands). hMSCs were isolated from the BM samples as described previously [3]. Briefly, the cells were seeded in Dulbecco's modified Eagle's medium (DMEM; Invitrogen-Gibco, Breda, the Netherlands) containing 100 U/ml penicillin (Invitrogen-Gibco), 100 µg/ml streptomycin (Invitrogen-Gibco) and 10% fetal bovine serum (FBS; Invitrogen-Gibco) in plastic cell culture flasks (Greiner Bio-One, Alphen aan den Rijn, the Netherlands) and incubated for 48 hours at 37°C in humidified air and 10% CO_2_. Thereafter, the non-attached cells were removed and the adherent cells were grown to 70% confluency before transfer for further expansion [3]. At passage number 2, aliquots of 2×10^5^ cells were cryopreserved. hMSC expansion was performed in culture medium supplemented with 0.5 ng/ml basic fibroblast growth factor (Sigma-Aldrich, St. Louis, MO). Cell replication was monitored for up to 15 passages from cultures initiated by seeding 5×10^4^/cm^2^ of freshly thawed cells in 25-cm^2^ cell culture flasks (Greiner bio-One, Frickenhousen, Germany). After reaching 70–80% confluency, the total number of cells in each culture was determined and a fraction of them was re-plated for a next passage under the same conditions as before.

The characterization of the cultured hMSCs by immunophenotyping and *in vitro* differentiation assays has been reported before [3],[66].

### Viral vectors and hMSC transduction

The coding sequences of the BHV-1 *UL49.5*, EBV *BNLF2a* and HCMV *US2* and *US11* genes were amplified from viral DNA by polymerase chain reaction (PCR). The resulting PCR fragments were inserted in the gammaretroviral vector shuttle plasmid pLZRS-IRES-GFP upstream of the internal ribosome entry site as described previously [35], [36], [67], [68]. Recombinant retroviruses directing the synthesis of eGFP and either of the four herpesviral immunoevasins were prepared using the Phoenix amphotropic packaging system as described previously [69].

Culture-expanded hMSCs (passages 4 to 8) were transduced with the recombinant retroviruses in RetroNectin (Takara Bio Europe, Saint-Germain-en-Laye, France)-coated dishes. Two or three rounds of transduction were performed at 24-hour intervals.

For *in vivo* studies, RV-transduced hMSCs were sorted on the basis of eGFP levels using a FACSDiva (Becton Dickinson [BD], Breda, the Netherlands). The eGFP^+^ MHC class I^-^ cells as well as untransduced hMSCs from the same batch of cells were then exposed to the vesicular stomatitis virus G protein-pseudotyped self-inactivating lentiviral vector LV.C-EF1a.cyt-bGal [70], encoding *Escherichia coli* β-gal. The cells were incubated for 4 hours with 2 HeLa cell-transducing units of the LV.C-EF1a.cyt-bGal vector/hMSC in DMEM containing 10% FBS and 8 µg/ml hexadimethrine bromide (polybrene; Sigma-Aldrich) at 37°C [71]. Transduction efficiencies were determined 14 days later by staining the cells with 2 mM 5-bromo-4-chloro-3-indolyl-β-D-galactopyranoside (Sigma-Aldrich) as described previously [72]. Typically, more than 90% of the LV.C-EF1a.cyt-bGal-transduced hMSCs were β-gal^+^ at this time point as well as after multiple *ex vivo* cell doublings.

### Antibodies and flow cytometry

Flow cytometric analyses were performed with a FACSort (BD) using the following murine monoclonal antibodies (MAbs): phycoerythrin (PE)-conjugated anti-human CD44 (clone G44-26; BD), PE-conjugated anti-HLA-ABC complex (clone W6/32; Dako Netherlands, Heverlee, Belgium) and PE-conjugated anti-HLA-DR (clone L243; BD). NK cells in the peripheral blood samples of the recipient mice were detected with PE-conjugated anti-mouse NK1.1 (clone PK126; BD), fluorescein isothiocyanate (FITC)-conjugated anti-mouse CD3 (clone 17A2; BD) and peridinin-chlorophyll-protein-complex (PerCP)-conjugated anti-mouse CD45 (clone 30-F11; BD) MAbs. Control MAbs (mouse IgGs conjugated with FITC, PE or PerCP; BD) were used for gate positioning.

### Immunoblotting

RV-transduced hMSCs were lysed in 0.5% Nonidet P-40, 50 mM Tris-HCl (pH 7.5), 5 mM MgCl_2_, 10 µM leupeptin (Sigma-Aldrich) and 1 mM 4-(2-aminoethyl) benzenesulfonyl fluoride (Sigma-Aldrich). The proteins in the lysates were separated by sodium dodecyl sulfate-polyacrylamide gel electrophoresis and transferred to polyvinylidene fluoride membranes (Hybond-P; GE Healthcare Europe, Diegem, Belgium). The resulting blots were incubated with the rat monoclonal antibody MVH 5B9 for the detection of BNLFA2a [73], a rabbit polyclonal antiserum directed against amino acids 22 through 51 of the BHV-1 UL49.5 protein [74] or a rabbit polyclonal antiserum specific for the N-terminus of the HCMV US2 protein (amino acids 24 through 41; [68]). For the detection of the HCMV US11 protein, a rabbit antiserum directed against the N-terminus of the protein was used (amino acids 21 through 38; [67]). Horseradish peroxidase-conjugated secondary antibodies (Dako Netherlands) and Amersham ECL Plus Western Blotting Detection Reagents (GE Healthcare Europe) were used to visualize bound primary antibodies.

### Mice and the xenotransplantation model

NOD/LtSz-scid/scid/J (NOD/SCID) mice were bred in the animal facilities of the LUMC from breeding pairs purchased from Jackson Laboratories (Bar Harbor, ME). Animals were housed under specific pathogen-free conditions as described previously [75].

C57Bl/6J (H-2^b^) (C57Bl) mice were purchased from Harlan (Venray, the Netherlands). Male and female mice 8–12 weeks old were used. The animal room was on a 12∶12-hours light-dark cycle and kept at 22°C. Standard laboratory chow and sterile water were provided *ad libitum*. All experiments were performed according to a study protocol approved by the Animal Ethics Committee of the LUMC.

hMSC engraftment was studied by subcutaneous injection of 3–5×10^5^ *LacZ*-tagged cells in a total volume of 20 µl phosphate-buffered saline (PBS) into the pinna of one of the ears of either NOD/SCID mice, C57Bl mice or C57Bl mice that had been depleted of NK cells by intraperitoneal injection of 100 µg per treatment of sodium azide-free affinity purified anti-mouse NK1.1 MAbs (clone PK136; BD or BioLegend, San Diego, CA). Antibody administration was initiated 24 hours prior to hMSC implantation and repeated once a week thereafter. The level of NK cells in these mice was determined by flow cytometric analysis of PBMCs that were sampled prior to each antibody injection and before sacrificing the mice. PBMCs of untreated mice served to derive control values.

The relative amount of *LacZ*-expressing human cells in the ears of the treated mice was determined with the Beta-Glo assay system (Promega Corporation, Madison, WI) [76], [77]. At different time points after hMSC implantation, mice were sacrificed, the whole treated ear was dissected, snap-frozen in liquid nitrogen and stored at −80°C until processing. Each ear was homogenized in 1 ml of homogenization buffer (10 mM KCl, 1.5 mM MgCl_2_ and 10 mM Tris-HCl [pH 7.4]) on ice as described [78]. The homogenate was cleared by centrifugation for 10 minutes at 20,800×G and 4°C. The supernatants were analyzed for β-gal activity according to the protocol provided by the manufacturer of the Beta-Glo assay system. Chemoluminescence was measured using a Wallace 1420 VICTOR 3 multilabel plate reader (PerkinElmer Nederland_,_ Groningen, the Netherlands). β-gal activity was expressed in RLUs. Serial dilutions of each supernatant were analyzed and plotted against RLUs. RLUs of different samples were compared at a dilution positioned in the linear range of the dose-response curve.

For each experimental group, the β-gal activity at each time point is presented as a percentage of the average β-gal activity detected in the ears of mice immediately (i.e. within 5 minutes) after cell injection (time 0; 100%), allowing a direct comparison of all experimental groups in the four *in vivo* experiments that were performed.

### Immunohistology of pinnea

C57Bl mice were injected as above with *LacZ*-tagged hMSCs. Three mice per treated group were sacrificed at 3, 7 and 14 days after cell injection. Ears were dissected and frozen by liquid nitrogen for one minute and stored at −80°C until further processing. Three consecutive transversal cross-sections of 8 µm were placed on SuperFrost Plus glass slides (Menzel-Gläser, Braunschweig, Germany) and stored at −20°C.

The first slide of each pinna was processed for X-gal staining as described before [70]. Sections were counterstained with nuclear fast red and saffron according to standard procedures and mounted in Entellan mounting medium (Merck, Schiphol-Rijk, the Netherlands).

Immunostaining of the tissues was performed with antibodies specific for CD8 and NK cells. The sections were fixed with ice-cold acetone (Mallinckrodt Baker, Deventer, the Netherlands) for 5 minutes at 4°C, air-dried and washed once for 5 minutes with PBS. A peroxidase blocking step was performed by incubating the sections for 10 minutes with 0.3% H_2_O_2_ in H_2_O followed by two PBS washes for 5 minutes each. Sections were then treated with the M.O.M. Immunodetection Kit Basic (Vector Laboratories, Burlington, CA) accordingly to the manufacturer's specifications. For the staining of CD8^+^ cells, biotinylated rat anti-mouse CD8a MAb (Biolegend; clone 53-6.7; IgG2a; 1∶250) was added for overnight incubation at 4°C. As isotype control antibody we used biotin-conjugated rat IgG2a MAb (Biolegend; clone RTK2758; 1∶200). Antibodies were removed by washing twice with PBS for 5 minutes. The Vectastain Elite ABC Kit (Vector Laboratories) was then applied according to manufacturer's specifications. Bound antibody complexes were visualized with 3,3′-diaminobenzidine (DAB, Sigma-Aldrich). Sections treated with DAB only were used to stain granulocytes. Finally, the sections were counter-stained with hematoxylin, dehydrated and mounted in Entellan mounting medium. Images were captured with a ColorView IIIu camera (Olympus Nederland, Zouterwoude, the Netherlands) mounted on a Leitz Wetzlar Diaplan microscope (Germany) and processed with Cell^B^ imaging software (Olympus).

Three antibodies were used for the detection of NK cells: the biotinylated mouse anti-mouse NK1.1 MAb (Biolegend; clone PK136; IgG2a; 1∶10), the goat anti-mouse NKp46 polyclonal antibody (R&D Systems Europe, Abingdon, United Kingdom; IgG; 1∶100) and the rat anti-mouse Ly49G2 MAb (BD; clone 4D11; IgG2a; 1∶20). The corresponding isotype controls were a mouse IgG2a MAb (Biolegend; clone MOPC-173; 1∶200), goat serum (1∶10) and the previously mentioned biotin-conjugated rat IgG2a MAb (1∶200). Tissues were incubated overnight at 4°C with the first antibody. After washing with PBS, tissues were exposed to a 2.5-hour incubation with streptavidine-conjugated Qdot 655 (Invitrogen-Molecular Probes; 1∶200), Alexa 594-coupled donkey anti-goat IgG (Invitrogen-Molecular Probes; 1∶800) or Alexa 488-conjugated donkey anti-rat IgG (Invitrogen-Molecular Probes; 1∶400), respectively. PBS-washed sections were then incubated with Hoechst 33342 (1∶1000 in PBS, Invitrogen-Molecular Probes) for 10 minutes, washed thoroughly (3–4 times) with PBS and mounted in Vectashieled. Microscopic analysis was performed on using Leica DM5500 B fluorescence microscope (Leica Microsystems, Rijswijk, the Netherlands). Images were captured with a cool SNAP K4 CCD camera (Photometrics, Tucscon, AZ) and archived with home-made software.

### Statistics

Data were analyzed with the one-way analysis of variance. Results are expressed as the mean ± standard deviation (SD). A p-value <0.05 was considered significant.
